# Associations between dynamic change of Chinese visceral adiposity index and hypertensive co-morbidities in the middle-aged and elderly population: a Chinese prospective cohort study

**DOI:** 10.3389/fnut.2025.1557868

**Published:** 2025-07-09

**Authors:** Jinjian Xu, Yingdi Yang, Ye Hu, Yingxiang Song, Xiao Ye, Yu-ming Chen, Hanbing Li, Xiaohong Wu

**Affiliations:** ^1^Key Laboratory of Endocrine Gland Diseases of Zhejiang Province, Department of Endocrinology, Geriatric Medicine Center, Zhejiang Provincial People’s Hospital (Affiliated People’s Hospital, Hangzhou Medical College), Hangzhou, China; ^2^Department of Clinical Medicine, Xinjiang Medical University, Urumqi, China; ^3^Department of Epidemiology, School of Public Health, Sun Yat-sen University, Guangzhou, China; ^4^Department of Pharmaceutical Sciences, Institute of Pharmacology, Zhejiang University of Technology, Hangzhou, China

**Keywords:** Chinese visceral adiposity index, trajectory analysis, hypertension, comorbidity, prospective cohort study

## Abstract

**Background and aims:**

The non-invasive Chinese visceral adiposity index (CVAI) was linked to the risk of cardiovascular disease and mortality. The associations of the CVAI and its longitudinal changes with hypertension and related comorbidities remain poorly understood. This study aims to examine the associations between CVAI, its trajectories, and hypertensive comorbidities.

**Methods:**

This study included 5,058 participants from the China Health and Retirement Longitudinal Study (CHARLS), with baseline data collected in 2011–2012. Participants were subsequently followed across four waves: 2013–2014, 2015–2016, 2017–2018, and 2019–2020. Study outcomes included hypertension (HTN) and its comorbidities: overweight (HTN-OW), metabolic unhealthiness (HTN-MET), and diabetes (HTN-DM). Prospective associations between baseline CVAI and these outcomes were analyzed using Cox regression. Longitudinal trajectories of CVAI over 3 years (2012–2015) were identified through K-means clustering analysis.

**Results:**

From 2013 to 2020, 1,581 participants (31.3% of the 5,058 total) developed HTN. The higher CVAI was significantly associated with risk of HTN (HR per 1 SD increase = 1.20; 95% CI: 1.08–1.33) and hypertensive co-morbidities (HTN-OW, HTN-MET, and HTN-DM) (HR = 1.20–1.38, *p* < 0.01). Both per-quartile increment in CVAI and cumulative CVAI showed significant positive associations with HTN risk and its comorbidities (all *p*-trend < 0.05). *K*-means clustering analysis generated three trajectories of change in CVAI levels (low, moderate, and high) between 2012 and 2015, and higher class was significantly associated with risk of HTN and co-morbidities compared to low class (HR = 1.63∼1.65, *p* < 0.05, *p*-trend < 0.05).

**Conclusion:**

The findings highlight the significant positive correlation between the change in CVAI with hypertension and comorbidities in the middle-aged and elderly population, suggesting a potential application in the clinical assessment and prevention of cardiometabolic disease.

## Introduction

Primary hypertension (HTN) affects nearly one-third of adults worldwide and is one of the leading causes of premature death (1). In 2015, 245 million Chinese adults over the age of 18 suffered from hypertension, and 2.54 million patients died from elevated systolic blood pressure (SBP) in 2017, with a disability-adjusted life expectancy of more than 5% (2). HTN has become a serious problem in public health due to the promoter for adverse cardiovascular events, kidney damage, and mortality (3, 4).

Visceral fat accumulation (VFA) significantly contributes to the risk of hypertension, metabolic syndrome and stroke in European and American populations (5), and is positively associated with cardiovascular disease and mortality in East Asian population (6). VFA was identified as a significant independent risk factor for cardiovascular and all-cause mortality (7). Although, imaging-based computed tomography (CT), magnetic resonance imaging (MRI) and dual energy X-ray absorptiometry (DEXA) can accurately visualize the distribution of visceral fat in abdomen, their unaffordable price, bulky equipment have limited the promotion and application in clinical and epidemiological investigations (8–10). Anthropometric measures and lipid levels have been used by the visceral adiposity index (VAI) to determine the quantity of visceral fat which can reflect the distribution of visceral fat in Western population (11, 12).

The Chinese visceral adiposity index (CVAI), an improved version of the VAI, integrates age, BMI, waist circumference, triglyceride levels, and HDL cholesterol levels to better match Chinese composition and was significantly associated with cardiovascular diseases (CVD), diabetic complications and mortality (13–16). CVAI was positively linked with carotid plaque risk in a non-linear dose-response pattern (17). In recent years, study found that both CVAI and other abdominal obesity indices were associated with cardiovascular events and highlighted that CVAI may be a valuable indicator for identifying Chinese T2DM patients at high risk of CVD (18). Moreover, elevated CVAI was substantially and favorably linked to the risk of T2DM in hypertensive patients (19). Conversely, one standard deviation (SD) increase in CVAI was not linked to the incidence of diabetic retinopathy (DR), but it was significantly linked to a higher prevalence of diabetic kidney disease (DKD) in both men and women (14). The previous studies were not clear about the prospective relationship between VAI or CVAI with HTN. Meanwhile, the relationship between the longitudinal change in CVAI and the risk of new-onset HTN needs to be further explored. Studies have shown that patients with HTN have a higher prevalence of co-morbidities with obesity, diabetes mellitus, and abnormal lipid metabolism (20). The presence of co-morbidities was associated with higher risk of mortality, and disability (21). However, studies on the association between CVAI and hypertensive co-morbidities were sparse.

In this nationwide prospective cohort study, we systematically evaluated the associations of baseline Chinese visceral adiposity index (CVAI) levels and their longitudinal trajectories with incident hypertension and its cardiometabolic comorbidities in Chinese adults.

## Materials and methods

### Study participants

**FIGURE 1:**
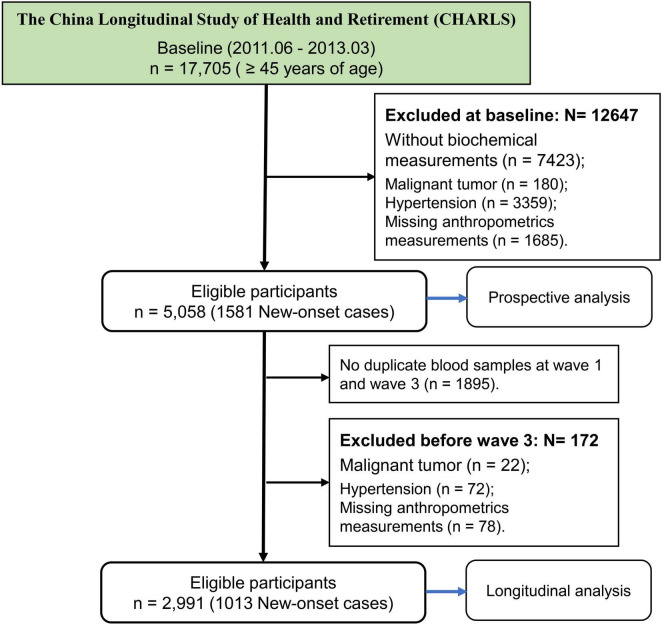
The flowchart of study participants.

The current study was based on a nationwide cohort of the China Health and Retirement Longitudinal Study (CHARLS)^1^ which aims to collect high-quality health data representing families and individuals of middle-aged and elderly people aged 45 and above in China, to illuminate the problem of aging in China, and promote interdisciplinary study. Participants are drawn from both rural and urban residence using a multistage stratified probability proportional-to-size sampling strategy, and there are five waves between 2011 and 2020 (22). The first survey (2011–2012) was used as a baseline, and then participants were followed up at four subsequent visits (2013–2014, 2015–2016, 2017–2018, 2019–2020). Totally, 17,705 participants were recruited at baseline. This study further selected the subjects at baseline according to the exclusion criteria: (1) without biochemical measurements (*n* = 7,423); (2) malignant tumor (*n* = 180); (3) hypertension and comorbidities (*n* = 3,359); (4) missing anthropometrics measurements (*n* = 1,685). Finally, 5,058 participants were eligible for further study (Figure 1).

The CHARLS study was approved by the institutional review board of Peking University. All participants provided written informed consent.

### Definition of outcomes

Primary hypertension (PHTN) was defined as blood pressure measurements with systolic blood pressure (SBP) ≥ 140 mmHg and/or a diastolic blood pressure (DBP) ≥ 90 mmHg on three readings or use of antihypertensive drugs (23). Diagnosis of type 2 diabetes mellitus defined as random blood glucose ≥ 11.1 mmol/L, or fasting blood glucose ≥ 126 mg/dL (7.0 mmol/L), or HbA1c ≥ 6.5%, or oral glucose tolerance test (OGTT) 2-h glucose ≥ 200 mg/dL (11.1 mmol/L), and/or classic symptoms of hyperglycemia, and/or hypoglycemic therapy (24). BMI was used to evaluate the state of obesity. Participants in the CHARLS were divided into normal weight (18.5 ≤ BMI < 24.0 kg/m^2^), overweight (24.0 ≤ BMI < 28.0 kg/m^2^), and obesity (BMI ≥ 28.0 kg/m^2^) (25). Metabolic status was assessed based on four metabolic components (26). Participants were categorized as metabolically unhealthy if they satisfied two or more of the four following criteria (27): (1) Abdominal obesity: WC ≥ 90 cm for men and WC ≥ 85 for women; (2) Impaired glycemic control: fasting plasma glucose (FPG) ≥ 5.6 mmol/L or use of antidiabetic drugs; (3) Elevated triglyceride (TG): TG ≥ 1.7 mmol/L or use of lipid-lowering drugs; (4) Reduced high-density lipoprotein cholesterol (HDL-C): HDL-C < 1.03 mmol/L for men or < 1.29 mmol/L for women or use of lipid-lowering drugs. According to the hypertension and coexisting conditions with overweight, metabolically unhealthy, or diabetes, the four phenotypes of hypertension and comorbidities were generated as HTN (Simple hypertension), HTN-OW (hypertension combined overweight), HTN-MET (hypertension combined metabolically unhealthy), and HTN-DM (hypertension combined diabetes).

### Data collection for covariates and exposures

The venous blood samples were taken at two waves (wave 1 and 3) by trained professionals using a standard phlebotomy process and tested for biochemical parameters. Following the separation of the venous blood into plasma and buffy coat, the plasma was kept in three 0.5 mL cryovials, while the buffy coat was kept in a different cryovial. After being instantly frozen at −20°C, these cryovials were shipped to the Chinese Center for Disease Control and Prevention (China CDC) in Beijing within 2 weeks (22). The blood samples from participants were tested for complete blood count (CBC) and blood chemistry panel. The lipid profiles and FPG were detected by enzymatic colorimetric test, while HbA1c was measured using boronate affinity high performance liquid chromatography. The methods and detection limits of bioassays were shown in Supplementary Table S1. Several covariates were collected by trained interviewers with standard questionnaires, including demographic information (such as age, gender, education, residence, living standard, marital status, smoking status, and alcohol consumption), health status and functioning (such as history of diseases and medication use). The detailed information about the categorical variables was presented in Supplementary material.

The primary exposures of this study were CVAI levels at baseline and change of CVAI between 2012 and 2015 (28). The minor exposures primarily involved common lipid metabolism indices, including body roundness index (BRI), lipid accumulation product (LAP), WC, WHtR, and BMI. The cumulative CVAI was determined by the expression: (CVAI_–2012_ + CVAI_–2015_)/ 2 × time _(2015–2012)_ (29). The equations and information for primary exposure of CVAI and minor exposures of BRI and LAP were provided in Supplementary Table S2.

### Statistical analysis

In the study, continuous variables were presented as means (SD), while categorical variables were reported as counts (percentages). Baseline characteristics between groups were compared using one-way ANOVA or the Kruskal-Wallis test for continuous variables, and the Pearson’s Chi-squared test for categorical variables.

The predictive abilities of CVAI and secondary exposures of adipose indices (BRI, LAP, WHtR, BMI, and WC) to risk of HTN and comorbidities were explored by receiver operator characteristic curve (ROC). In prospective study, restricted cubic spline (RCS) curve with 4 knots was conducted to test non-linearity for CVAI when estimating HTN, HTN-OW, HTN-MET, and HTN-DM. Then, Cox model assumption of proportionality was checked for CVAI and secondary exposures of adipose indices (BRI, LAP, WHtR, BMI, and WC). The associations of adipose indices with HTN and comorbidities were quantified using hazard ratios (HRs) with 95% CIs before and after adjusting for confounding factors. Crude model, didn’t adjust any covariable; Model 1 was adjusted for sex and age; Model 2 was adjusted for sex, age, (BMI), education, marital status, residence, living standard, smoking status, drinking status, uric acid, serum creatinine, FPG, history and medication use of CVD, liver disease, kidney disease, chronic lung diseases.

In the longitudinal studies for the associations of cumulative CVAI with HTN and comorbidities incidences after wave 3 were conducted by Cox proportional-hazards regression. The longitudinal trajectory of CVAI within 3 years was investigated using *K*-means clustering analysis, a form of unsupervised machine learning technology. We utilized the elbow approach to determine the optimal number of clusters (30). Each observation was assigned to the cluster with the closest mean value, which serves as the prototype of the cluster. The relationships between longitudinal trajectories of CVAI with HTN and comorbidities were assessed using Cox proportional-hazards regression in three models with the same adjustment of covariates as above. Meanwhile, the subgroup analyses of CVAI and trajectories in relation to HTN and its co-morbidities across multiple categories of confounders and disease states were performed with Cox regression models, and the interaction effects of CVAI with confounders in relation to HTN and its co-morbidities were also explored.

All analyses were performed with the R programming environment version 4.4.0 (R Project for Statistical Computing). *P*-values were 2-tailed, with *P* < 0.05 indicating statistical significance.

## Results

### The design of study

**FIGURE 2:**
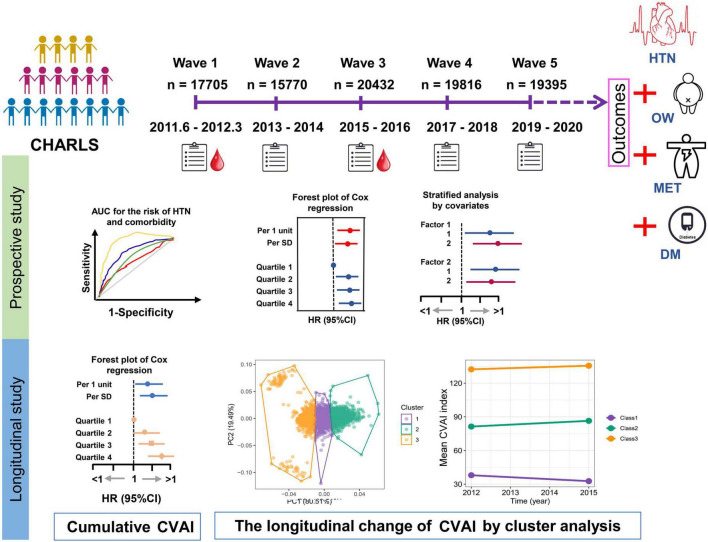
The design of study. The current study was based on a nationwide cohort of the China Health and Retirement Longitudinal Study (CHARLS) which the first survey (2011–2012) was used as a baseline, and then participants were followed up at four subsequent visits (2013–2014, 2015–2016, 2017–2018, and 2019–2020). The HTN and coexisting conditions with overweight (HTN-OW), metabolically unhealthy (HTN-MET), or diabetes (HTN-DM) were study outcomes. The prospective associations of baseline Chinese visceral fat index (CVAI) and other adipose indices with HTN and comorbidities were estimated by Cox regression. In the longitudinal studies for the associations of cumulative CVAI and other adipose indices with HTN and comorbidities incidences after wave 3 were conducted by Cox regression. The longitudinal trajectory of the change in CVAI within 3 years (2012∼2015) was investigated using *K*-means clustering analysis.

The current study was based on a nationwide cohort of the China Health and Retirement Longitudinal Study (CHARLS), in which participants were followed up at four successive visits (2013–2014, 2015–2016, 2017–2018, and 2019–2020) after the initial survey (2011–2012) served as a baseline. The outcomes of the study included hypertension (HTN) and coexisting conditions with diabetes (HTN-DM), metabolically unhealthy (HTN-MET), or overweight (HTN-OW). The primary exposure of study was CVAI level at baseline and change of CVAI between 2012 and 2015 (Figure 2).

### Baseline characteristics of study participants

A total of 5,058 study participants were included in this study, with 1,581 patients with new-onset essential hypertension from 2013 to 2020. The baseline clinical and demographic characteristics of participants were grouped by quartiles of CVAI and longitudinal change trajectories in CVAI. The means (SD) of age and BMI at baseline was 57.03 (9.24) years and 22.62 (3.25) kg/m^2^, with 2,679 (53%) being female. Participants with higher CVAI quartiles and groups of trajectories tended to be higher age, BMI, WC, WHtR, SBP, DBP, FPG, and lipids profiles compared to those in the lowest quartile and trajectory class (*p* < 0.001) (Table 1, Supplementary Table S3).

**TABLE 1:** Baseline characteristics of study participants by the quartiles of CVAI.

Characteristic	Overall	Quartile 1	Quartile 2	Quartile 3	Quartile 4	*p*-value^a^
	*N* = 5,058	*N* = 1,603	*N* = 1,433	*N* = 1,215	*N* = 807	
Age (years)	57.03 (9.24)	54.91 (8.73)	56.75 (8.96)	57.92 (9.23)	60.42 (9.57)	<0.001
Sex, *n* (%)						<0.001
Female	2,679 (53%)	730 (46%)	807 (56%)	742 (61%)	400 (50%)	
Height (cm)	158.09 (8.26)	158.16 (7.57)	157.62 (8.29)	157.50 (8.54)	159.67 (8.89)	<0.001
Weight (kg)	56.69 (9.98)	50.65 (7.26)	54.87 (7.71)	59.50 (8.43)	67.70 (9.76)	<0.001
BMI (kg/m^2^)	22.62 (3.25)	20.20 (2.20)	22.03 (2.24)	23.93 (2.48)	26.51 (2.93)	<0.001
WC (cm)	81.74 (11.58)	71.72 (11.55)	80.61 (5.94)	87.03 (5.04)	95.69 (6.07)	<0.001
WHtR	0.52 (0.08)	0.45 (0.07)	0.51 (0.04)	0.55 (0.04)	0.60 (0.05)	<0.001
SBP (mmHg)	118.12 (11.62)	115.61 (11.73)	117.46 (11.74)	119.55 (10.92)	122.14 (10.77)	<0.001
DBP (mmHg)	70.20 (8.84)	68.75 (8.88)	69.77 (8.77)	71.13 (8.63)	72.45 (8.63)	<0.001
MAP (mmHg)	86.18 (9.02)	84.37 (9.16)	85.67 (9.05)	87.27 (8.53)	89.01 (8.46)	<0.001
Heart rate (bpm)	71.95 (10.17)	71.02 (10.52)	71.43 (9.84)	72.78 (9.79)	73.49 (10.32)	<0.001
Education, *n* (%)						0.400
Primary school or lower	3,445 (68%)	1,080 (67%)	1,007 (70%)	822 (68%)	536 (66%)	
Middle school	1,083 (21%)	358 (22%)	283 (20%)	255 (21%)	187 (23%)	
Senior high school or higher	529 (10%)	164 (10%)	143 (10.0%)	138 (11%)	84 (10%)	
Smoking, *n* (%)						<0.001
Never	3,051 (60%)	854 (53%)	898 (63%)	822 (68%)	477 (59%)	
Ever	391 (7.7%)	101 (6.3%)	93 (6.5%)	90 (7.4%)	107 (13%)	
Current	1,616 (32%)	648 (40%)	442 (31%)	303 (25%)	223 (28%)	
Drinking, *n* (%)						0.700
Never	4,408 (87%)	1,394 (87%)	1,254 (88%)	1,068 (88%)	692 (86%)	
Ever	246 (4.9%)	77 (4.8%)	62 (4.3%)	60 (4.9%)	47 (5.8%)	
Current	404 (8.0%)	132 (8.2%)	117 (8.2%)	87 (7.2%)	68 (8.4%)	
Living standard, *n* (%)						<0.001
High	112 (2.2%)	29 (1.8%)	23 (1.6%)	30 (2.5%)	30 (3.8%)	
Average	2,638 (53%)	755 (48%)	781 (55%)	675 (56%)	427 (54%)	
Poor	2,245 (45%)	795 (50%)	612 (43%)	498 (41%)	340 (43%)	
Residence, *n* (%)						<0.001
Urban	4,694 (93%)	1,525 (95%)	1,337 (93%)	1,109 (91%)	723 (90%)	
Rural	364 (7.2%)	78 (4.9%)	96 (6.7%)	106 (8.7%)	84 (10%)	
Marital, *n* (%)						<0.001
Married	4,572 (90%)	1,478 (92%)	1,300 (91%)	1,095 (90%)	699 (87%)	
Other	486 (9.6%)	125 (7.8%)	133 (9.3%)	120 (9.9%)	108 (13%)	
WBC (10^9^/L)	6.12 (1.84)	6.09 (1.91)	6.06 (1.84)	6.17 (1.79)	6.22 (1.74)	0.009
MCV	90.49 (9.07)	90.63 (9.32)	90.32 (9.89)	90.27 (8.39)	90.83 (7.96)	0.300
HCT (%)	41.00 (6.31)	40.68 (5.94)	40.68 (6.50)	40.92 (6.43)	42.35 (6.35)	<0.001
Platelet (10^9^/L)	162.77 (38.42)	163.78 (37.82)	163.94 (38.30)	160.26 (39.08)	162.37 (38.77)	0.200
Cystatin C (mg/L)	1.02 (0.24)	1.01 (0.21)	1.01 (0.23)	1.02 (0.30)	1.06 (0.23)	<0.001
BUN (mg/L)	15.61 (4.50)	15.94 (4.64)	15.47 (4.71)	15.43 (4.37)	15.50 (3.99)	0.003
Scr (mg/dL)	0.87 (0.19)	0.86 (0.13)	0.87 (0.24)	0.88 (0.23)	0.89 (0.15)	0.013
Hemoglobin (g/dL)	14.19 (2.16)	14.03 (2.03)	14.07 (2.14)	14.15 (2.23)	14.74 (2.27)	<0.001
CRP (mg/L)	3.80 (8.63)	4.42 (11.71)	4.16 (8.82)	2.97 (5.03)	3.63 (7.16)	0.066
UA (mg/dL)	4.26 (1.17)	4.19 (1.16)	4.15 (1.12)	4.26 (1.18)	4.61 (1.20)	<0.001
FPG (mg/dL)	102.69 (18.57)	100.19 (17.22)	101.45 (17.73)	104.98 (19.23)	106.50 (20.58)	<0.001
TC(mg/dL)	176.38 (25.32)	174.72 (25.86)	175.69 (25.09)	177.99 (25.07)	178.84 (24.65)	<0.001
TG(mg/dL)	101.50 (42.47)	81.38 (34.23)	97.78 (38.18)	113.26 (41.44)	130.39 (44.05)	<0.001
LDL-C (mg/dL)	115.72 (31.23)	109.20 (29.27)	114.68 (30.77)	120.62 (31.84)	123.11 (32.09)	<0.001
HDL-C (mg/dL)	53.82 (14.89)	60.86 (16.24)	54.78 (13.12)	49.81 (12.31)	44.18 (10.71)	<0.001
HbA1c (%)	5.19 (0.70)	5.12 (0.63)	5.16 (0.68)	5.24 (0.77)	5.33 (0.76)	<0.001
eGFR (mL/min ⋅ 1.73 m^2^)	92.86 (14.07)	96.00 (12.70)	92.72 (13.81)	90.87 (14.46)	90.07 (15.17)	<0.001
CVAI	79.73 (39.22)	39.28 (30.85)	75.26 (7.11)	100.36 (8.01)	136.95 (19.05)	<0.001
BRI	3.81 (1.34)	2.64 (0.99)	3.63 (0.74)	4.45 (0.82)	5.49 (1.07)	<0.001
LAP	27.91 (21.03)	10.98 (13.66)	24.18 (11.20)	36.86 (14.26)	54.69 (21.01)	<0.001
TyG	8.45 (0.48)	8.22 (0.44)	8.42 (0.44)	8.60 (0.43)	8.77 (0.43)	<0.001
TyG-WC	691.73 (111.86)	588.61 (95.37)	678.46 (52.28)	748.25 (49.62)	838.56 (64.48)	<0.001
TyG-BMI	191.50 (31.96)	165.87 (19.83)	185.38 (19.90)	206.06 (22.93)	232.34 (28.12)	<0.001
TyG-WHtR	4.38 (0.72)	3.73 (0.62)	4.31 (0.37)	4.76 (0.37)	5.26 (0.47)	<0.001
CVD, *n* (%)	444 (8.8%)	119 (7.4%)	108 (7.6%)	111 (9.2%)	106 (13%)	<0.001
Liver disease, *n* (%)	187 (3.7%)	59 (3.7%)	51 (3.6%)	38 (3.1%)	39 (4.9%)	0.200
Kidney disease, *n* (%)	272 (9.2%)	94 (9.9%)	75 (9.5%)	58 (8.3%)	45 (8.4%)	0.600
Chronic lung diseases, *n* (%)	529 (10%)	187 (12%)	150 (11%)	112 (9.2%)	80 (10.0%)	0.200

*^a^*Kruskal-Wallis rank sum test; Pearson’s Chi-squared test. BMI, body mass index; WC, waist circumference; WHtR, waist-to-height ratio; SBP, systolic blood pressure; DBP, diastolic blood pressure; MAP, mean Arterial Pressure; WBC, white blood cell count; MCV, mean corpuscular volume; HCT, Hematocrit; BUN, blood urea nitrogen; Scr, serum creatinine; CRP, C reactive protein; UA, uric acid; FPG, fasting plasma glucose; TC, total cholesterol; TG, triglyceride; LDL-C, low-density lipoproteins cholesterol; HDL-C, high-density lipoproteins cholesterol; HBA1c, hemoglobin A1c; eGFR, estimated glomerular filtration rate; CVAI, Chinese visceral adiposity index; BRI, body roundness index; LAP, lipid accumulation product; TyG, triglyceride-glucose index; TyG-WC, triglyceride-glucose waist circumference; TyG-BMI, triglyceride-glucose body mass index; TyG-WHtR, triglyceride-glucose waist-to-height ratio; CVD, cardiovascular diseases.

Pearson’s correlation showed that CVAI was strongly related to BRI, LAP, WHtR, WC, BMI (*p* < 0.001), and the correlation coefficients of CVAI with BRI, WHtR, WC were greater than 0.8 (Supplementary Figure S1).

### Baseline CVAI with risk of hypertension and comorbidities

The ROC curves and area under the curves (AUCs) were used to predict the risk of hypertension (HTN) and comorbidities by baseline CVAI and other adipose indices.

**FIGURE 3:**
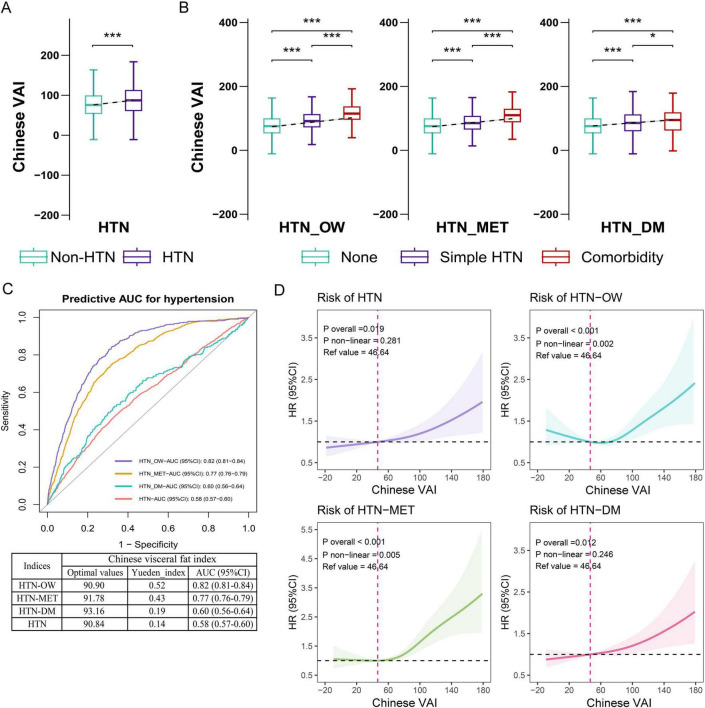
The associations of baseline CVAI with the risk of hypertension and comorbidities. **(A)** Difference in CVAI across status of hypertension. **(B)** Difference in CVAI across status of hypertension and comorbidities. **(C)** The ROC curves and area under the curves (AUCs) (95% CIs) of baseline CVAI for predicting the risk of hypertension and comorbidities. **(D)** RCS plot of the dose-response relationship of CVAI with hypertension and comorbidities. CVAI, Chinese visceral adiposity index; HTN, hypertension; HTN-OW, hypertension and overweight comorbidity; HTN-MET, hypertension and metabolic disorder comorbidity; HTN-DM, hypertension and diabetes comorbidity; ROC, receiver operator characteristic curve; RCS, restricted cubic spline.

**FIGURE 4:**
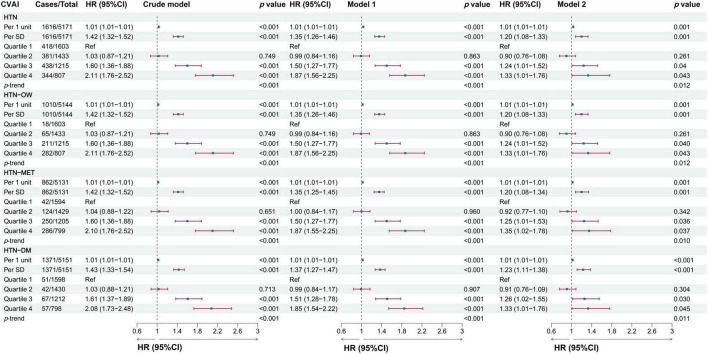
The associations between baseline CVAI and the risk of hypertension and comorbidities in different models. Crude model, didn’t adjust any covariable; Model 1, adjusted for sex and age; Model 2, adjusted for sex, age, BMI, education, marital status, residence, living standard, smoking status, drinking status, uric acid, serum creatinine, FPG, history of dyslipidemia, diabetes, CVD, liver disease, kidney disease, chronic lung diseases at baseline.

The results showed that the predictability of baseline CVAI to HTN, HTN-OW, HTN-MET, and HTN-DM was better compared to BRI, LAP, and WHtR (Supplementary Figures S2A–D). CVAI levels were significantly higher in the group with hypertension and hypertensive co-morbidities compared to those without HTN and co-morbidities (Figures 3A, B). Meanwhile, CVAI showed superior predictability to HTN-OW (AUC = 0.82) and HTN-MET (AUC = 0.77) risk (Figure 3C). A higher CVAI was significantly associated with higher risk of HTN, HTN-OW, HTN-MET, and HTN-DM (*p* < 0.05) in RCS study, and the significant non-linear relationships of CVAI with risk of HTN-OW (*p*-non-linear = 0.002) and HTN-MET (*p*-non-linear = 0.005) were observed (Figure 3D). Furthermore, CVAI was significantly associated with risk of HTN (HR per 1 SD increase = 1.20; 95% CI: 1.08–1.33) and hypertensive co-morbidities (HTN-OW, HTN-MET, and HTN-DM) (HR = 1.20–1.23, *p* < 0.01). The per-quartile increment in CVAI showed significant positive associations with HTN risk and its comorbidities (all *p*-trend < 0.05) (Figure 4).

### The longitudinal associations of change in CVAI with hypertension and comorbidities

**FIGURE 5:**
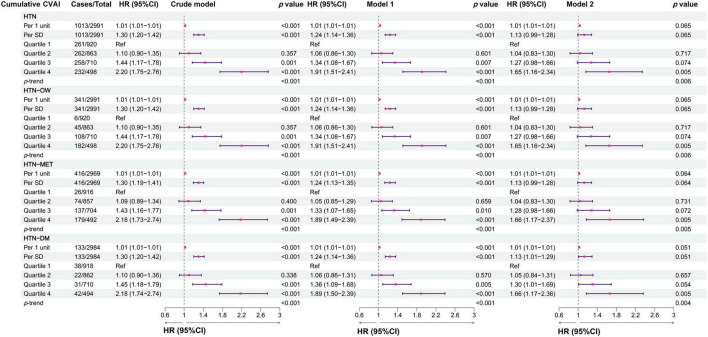
The associations between cumulative CVAI and the risk of hypertension and comorbidities in different models. Crude model, didn’t adjust any covariable; Model 1, adjusted for sex and age; Model 2, adjusted for sex, age, BMI, education, marital status, residence, living standard, smoking status, drinking status, uric acid, serum creatinine, FPG, history of dyslipidemia, diabetes, CVD, liver disease, kidney disease, chronic lung diseases at baseline.

**FIGURE 6:**
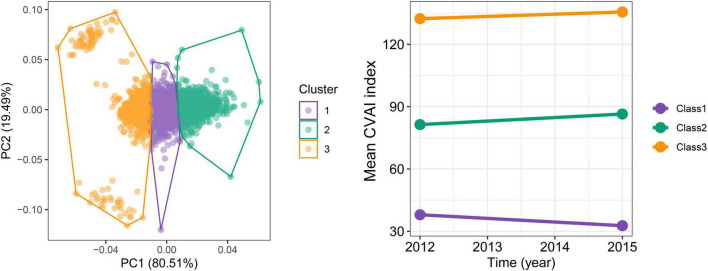
The trajectories for the changes of CVAI during follow-up periods by *K*-means clustering. The optimal numbers of clusters are determined using the elbow method by calculating the sum of squared errors.

**FIGURE 7:**
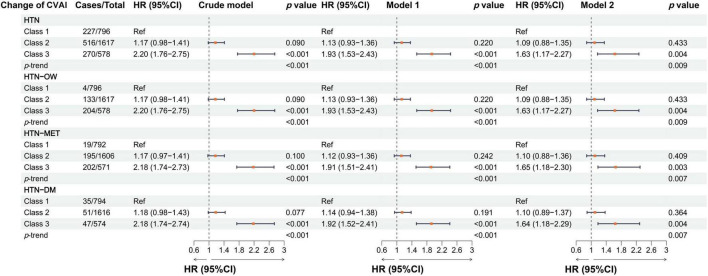
The associations between the longitudinal changes of CVAI and the risk of hypertension and comorbidities in different models. Crude model, didn’t adjust any covariable; Model 1, adjusted for sex and age; Model 2, adjusted for sex, age, BMI, education, marital status, residence, living standard, smoking status, drinking status, uric acid, serum creatinine, FPG, history of dyslipidemia, diabetes, CVD, liver disease, kidney disease, chronic lung diseases at baseline.

In the longitudinal study, we calculated cumulative CVAI using retested CVAI levels at baseline and wave3, and Cox regression found the 13% risk in HTN, HTN-OW, HTN-MET, and HTN-DM for each SD increase in cumulative CVAI. Meanwhile, the risk of HTN and co-morbidities increased by 65% with each quartile increment cumulative CVAI (*p*-trend < 0.05) (Figure 5). *K*-clustering analysis generated three trajectories of change in CVAI levels (low, moderate, and high) between 2012 and 2015, and higher class was positively associated with higher risk of HTN and co-morbidities compared to low class (HR = 1.63∼1.65, *p* < 0.05, *p*-trend < 0.05) (Figures 6, 7).

### Subgroup analyses

In the subgroup analyses stratified by sex, the associations between CVAI and HTN risk were both significant in males and females, and the HR was larger in females than in males [per 1-SD increase, for males: HR, 1.25 (95% CI, 1.15–1.37); for females: HR, 1.75 (95% CI, 1.55–1.97)]. We found a significant interaction effect between gender and CVAI in relation to HTN (*p*-interaction = 0.001). There was also a significant interaction effect between smoking and CVAI in relation to HTN (*p*-interaction = 0.002) (Supplementary Table S4). Moreover, the findings showed that the longitudinal change in CVAI was also a significant interaction with sex in relation to HTN (*p*-interaction = 0.036) (Table 2).

**TABLE 2:** Stratified associations between different classes of change in CVAI and hypertension incidence.

Subgroup	Change in the CVAI, OR (95% CI)			*P* for interaction
	**Class 1**	**Class 2**	**Class 3**	
Sex				0.001
Male	Ref	0.97 (0.75–1.24)	1.63 (1.20–2.21)**	
Female	Ref	1.65 (1.23–2.21)***	3.38 (2.39–4.77)***	
Age				0.732
<60	Ref	1.22 (0.97–1.54)	2.19 (1.62–2.96)***	
≥60	Ref	1.04 (0.76–1.43)	1.76 (1.23–2.50)**	
BMI, kg/m^2^				0.449
<24	Ref	1.13 (0.93–1.38)	2.72 (1.85–3.98)***	
≥24	Ref	2.24 (0.74–6.76)	3.70 (1.23–11.11)*	
Residence				0.036
Urban	Ref	1.17 (0.97–1.42)	2.17 (1.72–2.73)***	
Rural	Ref	1.57 (0.58–4.23)	3.76 (1.32–10.72)*	
Marital status				0.465
Married	Ref	1.18 (0.97–1.43)	2.19 (1.73–2.78)***	
Other	Ref	1.11 (0.58–2.10)	2.04 (0.97–4.28)	
Alcohol consumption				0.751
No	Ref	1.20 (0.99–1.46)	2.25 (1.78–2.84)***	
Yes	Ref	0.89 (0.46–1.73)	1.69 (0.77–3.72)	
Current smoking				0.133
No	Ref	1.55 (1.21–1.99)***	3.09 (2.31–4.12)***	
Yes	Ref	0.85 (0.64–1.15)	1.33 (0.90–1.98)	
Living standard				0.869
High	Ref	1.59 (0.41–6.23)	2.75 (0.61–12.41)	
Average	Ref	1.14 (0.88–1.49)	2.04 (1.48–2.80)***	
Poor	Ref	1.24 (0.95–1.63)	2.40 (1.73–3.34)***	
Educational level				0.401
Primary school or lower	Ref	1.18 (0.95–1.47)	2.15 (1.64–2.80)***	
Middle school	Ref	1.16 (0.77–1.76)	2.51 (1.53–4.11)***	
Senior high school or higher	Ref	1.18 (0.60–2.33)	2.10 (0.96–4.58)	
CKD				0.977
No	Ref	1.16 (0.89–1.50)	2.42 (1.79–3.28)***	
Yes	Ref	1.02 (0.48–2.16)	1.09 (0.40–2.95)	
CVD				0.833
No	Ref	1.17 (0.97–1.43)	2.27 (1.79–2.88)***	
Yes	Ref	1.24 (0.65–2.38)	1.65 (0.81–3.35)	

*P < 0.05; **P < 0.01; ***P < 0.001.

### Baseline adiposity indices and risk of hypertension and comorbidities

The risk of hypertension (HTN) and comorbidities was significantly related to the increasing quartiles of BRI, LAP, WC, WHtR, and BMI. HTN risk was also increased with per-SD increase in BRI, LAP, WC, WHtR, and BMI, the corresponding HRs for HTN with per-SD increase were 1.17 (95% CI, 1.07–1.27) for BRI, 1.15 (95% CI, 1.06–1.25) for LAP, 1.13 (95% CI, 1.05–1.21) for WC, 1.13 (95% CI, 1.05–1.22) for WHtR, 1.36 (95% CI, 1.28–1.44) for BMI (Supplementary Figure S3).

## Discussion

The current study found that the CVAI levels and its longitudinal trajectories were significantly positively associated with HTN and its co-morbidities in a middle-aged and elderly Chinese population. The higher CVAI and trajectory class were associated with higher risks of HTN and co-morbidities, and CVAI was non-linearly associated with the risk of HTN-OW and HTN-MET over the nearly 8-year follow-up period, and these associations were independent of BMI. The CVAI exerted a superior predictability to the risk of HTN-OW and HTN-MET over the future follow-up period. Meanwhile, the findings suggested that CVAI was in a stable dynamic between 2012 and 2015, and that the risks of HTN and co-morbidities were higher in the persistently high-level CVAI compared to the low-level population.

The VAI was created using an empirical mathematical model based on the BMI and WC, as well as lipid levels of HDL-C, and TG, which was strongly related to visceral fat distribution and validated through CT scans (12). Meanwhile, the CVAI was more correlated with HTN, T2DM, and CVD than traditional obesity indicators such as BMI, WC, and WHtR (16). In the HTN study, it was found that per 1 SD increase in CVAI after adjusting for confounders, the risks of HTN were over by 9% and 14% in men and women, respectively. The CVAI had a better predictive ability than both WC and BMI in hypertension (31). In addition, compared to the lowest quartile group, the highest quartile group of modified CVAI had a 1.747-fold higher risk of prehypertension and a 2.475-fold higher risk of hypertension in a cross-sectional study involving over 30,000 participants (32). However, studies have not explored the trajectory of CVAI in relation to HTN risk and have not examined the relationship with more complex HTN co-morbidities. Our study explored their prospective and longitudinal associations with HTN and its comorbidities using baseline CVAI and two repeated-measures trajectories of CVAI change, respectively. Those findings provided a better understanding to the association between CVAI and HTN in terms of temporal change.

In addition, CVAI was significantly associated with advanced cardiovascular events in Chinese population, with better predictive ability than LAP, VAI, WC, and BMI (33). Participants in quartiles (Q) 1-4 of CVAI had stroke occurrences of 4.42, 7.29, 9.06, and 13.04%, respectively. According to the fully adjusted model, the risk of incident stroke increases significantly with each 1.0-SD increase in CVAI (34). Poor glycemic control, dyslipidemia, and raised triglyceride-glucose index and its derivatives were observed in female T2DM patients with elevated VAI, indicating that VAI is a strong predictor of glycemic control status (35). Therefore, VAI and CVAI can offer crucial information for early cardiovascular disease screening and intervention as an indication that reflects the role of visceral adiposity.

Visceral fat was linked to insulin resistance and hyperinsulinemia, and was considered as an important indicator for advanced CVD (36). The alterations in hormone levels may affect renal sodium reabsorption and sympathetic nerve activity, thereby leading to elevated blood pressure (37). Additionally, visceral fat was also related to the secretion of epinephrine and norepinephrine, hormones that can cause vasoconstriction and increase blood pressure (38). Visceral adipose tissue was considered an important endocrine organ that secretes a variety of cytokines and adipokines, such as tumor necrosis factor-α (TNF-α) and interleukin-6 (IL-6) (39). These inflammatory mediators can trigger a systemic inflammatory response that leads to vascular endothelial dysfunction and promotes atherosclerosis, thereby increasing the risk of hypertension (40).

### Study strengths and limitations

The current study exhibits several noteworthy strengths that enhance its contributions to the understanding of the relationship between the CVAI and hypertension. Firstly, the study not only examines the relationship between CVAI and hypertension but also explores its association with various comorbidities, including HTN-OW, HTN-MET, and HTN-DM. This multi-faceted approach provides a holistic view of how visceral adiposity influences overall cardiovascular health, making the findings particularly relevant for comprehensive patient care. Additionally, the longitudinal design, with multiple follow-up assessments, allows for the examination of temporal changes in CVAI and strengthens causal inferences about its association with hypertension and its comorbidities. Furthermore, the focus on a middle-aged and elderly population addresses a critical demographic at heightened risk for hypertension and related conditions. The findings underscore the potential clinical utility of CVAI as a biomarker for risk assessment and intervention strategies aimed at preventing cardiometabolic diseases.

Despite these strengths, the study has limitations. The reliance on self-reported data may introduce recall bias, affecting result validity. Additionally, while the sample is substantial, its specific demographic context may limit generalizability to other populations, as lifestyle and genetic factors can vary. The association of CVAI with hypertension and its cardiometabolic complications, as well as its predictive efficacy, requires further replication and validation in diverse populations with varying ethnicities, dietary habits, and climatic backgrounds. Furthermore, the study may not adequately control unmeasured confounders, such as psychosocial and environmental influences. Lastly, focusing solely on CVAI as a predictor may overlook interactions with other adiposity measures.

## Conclusion

In conclusion, the current study elucidated the prospective associations between CVAI and its trajectories with HTN and the comorbidities among the middle-aged and elderly adults in China. Higher levels of CVAI were positively associated with an increased risk of HTN and comorbidities, and their association may also remain consistent across time-series changes. These results underscore the potential utility of CVAI as a critical tool for clinical assessment and risk stratification in the management of HTN and comorbidities. Nevertheless, it is imperative that future research endeavors validate these findings across diverse populations and further investigate the underlying mechanisms linking CVAI to HTN and its comorbidities.

## Data Availability

The datasets presented in this study can be found in online repositories. The names of the repository/repositories and accession number(s) can be found in the article/Supplementary material.

## Footnotes

1
http://charls.pku.edu.cn/
