# Supplementary material for: Associations between dynamic change of Chinese visceral adiposity index and hypertensive co-morbidities in the middle-aged and elderly population: a Chinese prospective cohort study
Source: Front Nutr. 2025 Jul 9;12:1557868. doi: 10.3389/fnut.2025.1557868 (PMC12283329; doi:10.3389/fnut.2025.1557868)
Supplement: Supplementary file 1 [file Table_1.docx]

**Supplementary materials**

Supplementary Methods

Supplementary Table S1 Methods, Coefficient of Variation, and Detection Limits of the Bioassays.

Supplementary Table S2 Equations of multiple cardiometabolic indices.

Supplementary Table S3 Baseline characteristics of study participants by the change of CVAI.

Supplementary Table S4 Stratified associations between baseline CVAI and hypertension incidence.

Supplementary Fig. S1 The correlation between anthropometric indices.

Supplementary Fig. S2 The ROC curves and area under the curves (AUCs) (95% CIs) of baseline CVAI for predicting the risk of hypertension and comorbidity.

Supplementary Fig. S3 Forest plot of the association between other adiposity indices and risk of hypertension and comorbidity.

**Supplementary Methods**

Education level is categorized into three groups: primary school or lower, middle school, and senior high school or higher. Residence is distinguished between rural and urban settings. Living standard is classified into three tiers: high, average, and poor. Marital status is divided into married and other statuses, which include divorced, separated, widowed, or single. Drinking status were never, ever (used to alcohol consumption but quit), current. Alcohol consumption is defined as drinking at least once per week. Smoking status were never, ever (used to smoke but quit), current (1). Participants, with the exception of those who had an arm injury, were given instructions to take three readings of their left arm blood pressure at 45-second intervals after a 15-minute rest. The average of the three readings was then reported. Before being weighed and having their height measured, participants were instructed to remove their shoes and clothing. Using standardized scales, measurements of height and body weight were made to the closest 0.1 cm and 0.1 kg, respectively.

| **Supplementary Table S1 Methods, Coefficient of Variation, and Detection Limits of the Bioassays** | | | | |
| --- | --- | --- | --- | --- |
| **Biomarkers** | **Method** | **Coefficient of variation** | | **Detection limits** |
|  |  | **Within assay** | **Between assay** |  |
| hsCRP | Immunoturbidimetric assay | <1.3% | <5.7% | 0.1-20 mg/L |
| HbAIc | Boronate affinity HPLC | 1.90% | 2.10% | 0-40% |
| TC | Enzymatic colormetric test | 0.80% | 1.70% | 3-800 mg/dL |
| HDL-C | Enzymatic colormetric test | 1.00% | 1.30% | 3-120 mg/dL |
| LDL-C | Enzymatic colormetric test | 0.70% | 1.20% | 3-400 mg/dL |
| TG | Enzymatic colormetric test | 1.50% | 1.80% | 4-1000 mg/dL |
| BUN | Enzymatic UV method with urease | <4.4% | <4.1% | 5-100 mg/dL |
| Scr | Rate-blanked and compensated Jaffe creatinine method | <1.6% | <2.1% | 0.1-25 mg/dL |
| FPG | Enzymatic colormetric test | 0.90% | 1.80% | 2-450 mg/dL |
| UA | UA Plus method | 1.10% | 1.90% | Up to 20 mg/dL |
| Cystatin C | Particle-enhanced turbimetric assay | <5% | <5% | 0.5-8 mg/L |

BUN, blood urea nitrogen; Scr, serum creatinine; hsCRP, high sensitivity C reactive protein; UA, uric acid; FPG, Fasting plasma glucose; TC, total cholesterol; TG, triglyceride; LDL-C, low-density lipoproteins cholesterol; HDL-C, high-density lipoproteins cholesterol; HBA1c, hemoglobin A1c.

**Supplementary Table S2 Equations of multiple cardiometabolic indices**

| **Cardiometabolic indices** | **Equation** |
| --- | --- |
| **CVAI (2; 3)** | Male: VAI=WC/(39.68+1.88×BMI) × TG/1.03×1.31/HDL-C  Female: VAI=WC/(36.58+1.89×BMI) × TG/0.81×1.52/HDL-C  Male: CVAI=-267.93+ 0.68×Age+0.03 ×BMI+ 4.00×WC+ 22.00×lgTG-16.32×HDL-C  Female: CVAI=-187.32+ 1.71×Age+4.23 ×BMI+ 1.12×WC+ 39.76×lgTG-11.66×HDL-C |
| **BRI (4)** | $BRI=364.2-365.5\times\sqrt{1-{(\frac{WC(cm)}{2\pi})}^{2}/{(0.5\times Height(cm))}^{2}}$ |
| **LAP (5)** | Male: LAP = (WC (cm)-61.3) × TG (mmol/L)  Female: LAP = (WC (cm)-55.6 × TG (mmol/L) |
| **TyG-WC (6)** | ln [TG (mg/dl) × glucose (mg/dl)/2] × WC (cm) |
| **TyG-WHtR (7)** | ln [TG (mg/dl) × glucose (mg/dl)/2] × WHtR |
| **TyG-BMI (8)** | Ln [TG (mg/dl) × glucose (mg/dl)/2] × BMI (kg/m^2^) |
| **TyG (9)** | Ln [TG (mg/dl) × glucose (mg/dl)/2] |

Abbreviations: CVAI, Chinese visceral adiposity index; BRI, body roundness index; TyG, triglyceride glucose index; LAP, lipid accumulation product; BMI, body mass index; FBG, fasting blood glucose (mmol/L); HDL-C, high-density lipoprotein cholesterol (mmol/L); TG, triglycerides (mmol/L); WHtR, waist-height ratio; WC, waist circumference (cm).

| **Supplementary Table S3 Baseline characteristics of study participants by the change of CVAI** | | | | | |
| --- | --- | --- | --- | --- | --- |
| **Characteristic** | **Overall** | **Class 1** | **Class 2** | **Class 3** | **p-value***^1^* |
|  | N = 2,991 | N = 796 | N = 1,617 | N = 578 |  |
| Age (years) | 57.08 (8.76) | 55.45 (8.83) | 56.87 (8.52) | 59.90 (8.68) | <0.001 |
| Sex, n (%) |  |  |  |  | <0.001 |
| Female | 1,577 (53%) | 330 (41%) | 946 (59%) | 301 (52%) |  |
| Height (cm) | 158.15 (8.03) | 157.99 (7.49) | 157.82 (8.09) | 159.29 (8.48) | 0.005 |
| Weight (kg) | 56.48 (9.51) | 50.02 (6.86) | 56.12 (7.77) | 66.40 (8.85) | <0.001 |
| BMI (kg/m^2^) | 22.54 (3.18) | 20.00 (2.05) | 22.50 (2.43) | 26.16 (2.84) | <0.001 |
| WC (cm) | 81.32 (11.75) | 71.57 (11.75) | 81.74 (8.07) | 93.57 (7.82) | <0.001 |
| WHtR | 0.52 (0.08) | 0.45 (0.08) | 0.52 (0.05) | 0.59 (0.05) | <0.001 |
| SBP (mmHg) | 117.83 (11.73) | 115.08 (11.92) | 117.75 (11.41) | 121.86 (11.22) | <0.001 |
| DBP (mmHg) | 69.96 (8.80) | 68.36 (8.99) | 69.90 (8.54) | 72.36 (8.75) | <0.001 |
| MAP (mmHg) | 85.92 (9.02) | 83.93 (9.33) | 85.85 (8.70) | 88.86 (8.68) | <0.001 |
| Heart rate (bpm) | 71.56 (9.87) | 70.89 (10.65) | 71.51 (9.46) | 72.65 (9.78) | <0.001 |
| Education, n (%) |  |  |  |  | 0.900 |
| Primary school or lower | 2,059 (69%) | 549 (69%) | 1,113 (69%) | 397 (69%) |  |
| Middle school | 640 (21%) | 174 (22%) | 344 (21%) | 122 (21%) |  |
| Senior high school or higher | 291 (9.7%) | 72 (9.1%) | 160 (9.9%) | 59 (10%) |  |
| Smoking, n (%) |  |  |  |  | <0.001 |
| Never | 1,817 (61%) | 398 (50%) | 1,054 (65%) | 365 (63%) |  |
| Ever | 220 (7.4%) | 62 (7.8%) | 92 (5.7%) | 66 (11%) |  |
| Current | 954 (32%) | 336 (42%) | 471 (29%) | 147 (25%) |  |
| Drinking, n (%) |  |  |  |  | 0.200 |
| Never | 2,607 (87%) | 696 (87%) | 1,418 (88%) | 493 (85%) |  |
| Ever | 133 (4.4%) | 34 (4.3%) | 63 (3.9%) | 36 (6.2%) |  |
| Current | 251 (8.4%) | 66 (8.3%) | 136 (8.4%) | 49 (8.5%) |  |
| Living standard, n (%) |  |  |  |  | 0.006 |
| High | 61 (2.1%) | 15 (1.9%) | 30 (1.9%) | 16 (2.8%) |  |
| Average | 1,585 (54%) | 382 (49%) | 902 (56%) | 301 (52%) |  |
| Poor | 1,314 (44%) | 387 (49%) | 669 (42%) | 258 (45%) |  |
| Residence, n (%) |  |  |  |  | 0.011 |
| Urban | 2,810 (94%) | 761 (96%) | 1,519 (94%) | 530 (92%) |  |
| Rural | 181 (6.1%) | 35 (4.4%) | 98 (6.1%) | 48 (8.3%) |  |
| Marital, n (%) |  |  |  |  | 0.086 |
| Married | 2,734 (91%) | 739 (93%) | 1,478 (91%) | 517 (89%) |  |
| Other | 257 (8.6%) | 57 (7.2%) | 139 (8.6%) | 61 (11%) |  |
| WBC (10^9^/L) | 6.10 (1.85) | 6.09 (1.86) | 6.06 (1.88) | 6.25 (1.74) | 0.007 |
| MCV | 90.62 (8.99) | 90.64 (9.50) | 90.45 (9.03) | 91.07 (8.12) | 0.500 |
| HCT (%) | 41.03 (6.28) | 40.76 (6.21) | 40.72 (6.41) | 42.26 (5.87) | <0.001 |
| Platelet (10^9^/L) | 163.60 (37.42) | 165.64 (36.71) | 163.22 (37.60) | 161.66 (37.92) | 0.300 |
| Cystatin C (mg/L) | 1.01 (0.22) | 1.01 (0.19) | 1.00 (0.24) | 1.02 (0.19) | 0.006 |
| BUN (mg/L) | 15.56 (4.27) | 16.03 (4.71) | 15.39 (4.14) | 15.41 (3.92) | 0.008 |
| Scr (mg/dL) | 0.86 (0.13) | 0.87 (0.12) | 0.85 (0.13) | 0.87 (0.12) | 0.007 |
| Hemoglobin (g/dL) | 14.20 (2.13) | 14.05 (1.96) | 14.13 (2.20) | 14.61 (2.11) | <0.001 |
| hsCRP (mg/L) | 3.56 (8.46) | 3.53 (7.94) | 3.90 (9.88) | 2.90 (4.91) | 0.800 |
| UA (mg/dL) | 4.19 (1.14) | 4.19 (1.16) | 4.10 (1.11) | 4.43 (1.16) | <0.001 |
| FPG (mg/dL) | 102.29 (17.94) | 101.14 (17.96) | 102.07 (17.81) | 104.52 (18.11) | <0.001 |
| TC (mg/dL) | 176.06 (25.20) | 174.25 (25.43) | 176.31 (25.29) | 178.00 (24.49) | 0.047 |
| TG (mg/dL) | 97.92 (40.59) | 81.38 (33.99) | 98.82 (39.48) | 118.20 (42.30) | <0.001 |
| LDL-C (mg/dL) | 114.65 (30.49) | 107.32 (28.71) | 115.94 (30.37) | 121.12 (31.27) | <0.001 |
| HDL-C (mg/dL) | 54.43 (14.89) | 61.28 (16.84) | 53.98 (13.37) | 46.27 (11.18) | <0.001 |
| HbA1c (%) | 5.19 (0.70) | 5.14 (0.64) | 5.17 (0.71) | 5.29 (0.73) | <0.001 |
| eGFR (mL/min·1.73 m^2^) | 93.81 (13.20) | 96.06 (12.56) | 93.38 (13.31) | 91.96 (13.39) | <0.001 |
| CVAI | 77.89 (38.52) | 37.98 (34.89) | 79.74 (17.68) | 127.64 (21.18) | <0.001 |
| BRI | 3.76 (1.33) | 2.64 (1.02) | 3.78 (0.96) | 5.23 (1.12) | <0.001 |
| LAP | 26.29 (20.14) | 11.14 (14.09) | 26.35 (15.13) | 47.00 (21.02) | <0.001 |
| TyG | 8.42 (0.46) | 8.23 (0.43) | 8.43 (0.45) | 8.64 (0.42) | <0.001 |
| TyG-WC | 685.02 (111.49) | 588.18 (100.41) | 689.13 (76.10) | 808.61 (78.03) | <0.001 |
| TyG-BMI | 189.94 (30.82) | 164.50 (18.94) | 189.73 (23.08) | 226.09 (27.32) | <0.001 |
| TyG-WHtR | 4.34 (0.72) | 3.73 (0.65) | 4.37 (0.51) | 5.09 (0.54) | <0.001 |
| CVD, n (%) | 253 (8.5%) | 60 (7.6%) | 120 (7.5%) | 73 (13%) | <0.001 |
| Liver disease, n (%) | 115 (3.9%) | 38 (4.8%) | 52 (3.2%) | 25 (4.4%) | 0.140 |
| Kidney diease, n (%) | 142 (8.2%) | 48 (10%) | 70 (7.7%) | 24 (6.6%) | 0.140 |
| Chronic lung diseases, n (%) | 311 (10%) | 102 (13%) | 155 (9.6%) | 54 (9.4%) | 0.033 |
| ^1^Kruskal-Wallis rank sum test; Pearson’s Chi-squared test. BMI, body mass index; WC, waist circumference; WHtR, waist-to-height ratio; SBP, systolic blood pressure; DBP, diastolic blood pressure; MAP, mean Arterial Pressure; WBC, white blood cell count; MCV, mean corpuscular volume; HCT, Hematocrit; BUN, blood urea nitrogen; Scr, serum creatinine; hsCRP, high sensitivity C reactive protein; UA, uric acid; FPG, Fasting plasma glucose; TC, total cholesterol; TG, triglyceride; LDL-C, low-density lipoproteins cholesterol; HDL-C, high-density lipoproteins cholesterol; HBA1c, hemoglobin A1c; eGFR, estimated glomerular filtration rate; CVAI, Chinese visceral adiposity index; BRI, body roundness index; LAP, lipid accumulation product; TyG, triglyceride-glucose index; TyG-WC, triglyceride-glucose waist circumferenc; TyG-BMI, triglyceride-glucose body mass index; TyG-WHtR, triglyceride-glucose waist-to-height ratio; CVD, cardiovascular diseases. | | | | | |

| **Supplementary Table S4 Stratified associations between baseline CVAI and hypertension incidence** | | | | | | | |
| --- | --- | --- | --- | --- | --- | --- | --- |
| **Subgroup** | **CVAI, HR (95% CI)** | | **Quartiles of CVAI, HR (95% CI)** | | | | ***P* for interaction** |
|  | **Per 1 unit** | **Per SD** | **Quartile 1** | **Quartile 2** | **Quartile 3** | **Quartile 4** |  |
| Sex |  |  |  |  |  |  | 0.001 |
| Male | 1.01 (1.01-1.01)^***^ | 1.25 (1.15-1.37)^***^ | Ref | 0.89 (0.71-1.12) | 1.46 (1.15-1.85)^**^ | 1.56 (1.22-2.00)^***^ |  |
| Female | 1.01 (1.01-1.02)^***^ | 1.75 (1.55-1.97)^***^ | Ref | 1.24 (0.98-1.56) | 1.87 (1.49-2.36)^***^ | 2.97 (2.28-3.86)^***^ |  |
| Age |  |  |  |  |  |  | 0.276 |
| <60 | 1.01 (1.01-1.01)^***^ | 1.41 (1.28-1.55)^***^ | Ref | 1.15 (0.94-1.40) | 1.59 (1.29-1.95)^***^ | 2.18 (1.71-2.77)^***^ |  |
| ≥60 | 1.01 (1.01-1.01)^***^ | 1.30 (1.16-1.45)^***^ | Ref | 0.75 (0.56-0.99)^*^ | 1.35 (1.03-1.78)^*^ | 1.53 (1.16-2.03)^**^ |  |
| BMI, kg/m2 |  |  |  |  |  |  | 0.364 |
| <24 | 1.01 (1.01-1.01)^***^ | 1.39 (1.25-1.55)^***^ | Ref | 1.04 (0.88-1.24) | 1.65 (1.35-2.01)^***^ | 2.10 (1.49-2.98)^***^ |  |
| ≥ 24 | 1.01 (1.01-1.01)^***^ | 1.46 (1.26-1.68)^***^ | Ref | 0.87 (0.47-1.62) | 1.37 (0.78-2.43) | 1.86 (1.05-3.28)^*^ |  |
| Residence |  |  |  |  |  |  | 0.411 |
| Urban | 1.01 (1.01-1.01)^***^ | 1.43 (1.33-1.55)^***^ | Ref | 1.02 (0.87-1.21) | 1.67 (1.42-1.98)^***^ | 2.09 (1.73-2.51)^***^ |  |
| Rural | 1.01 (1.01-1.02)^**^ | 1.46 (1.10-1.94)^**^ | Ref | 1.32 (0.61-2.85) | 1.16 (0.54-2.51) | 3.24 (1.54-6.78)^**^ |  |
| Marital status |  |  |  |  |  |  | 0.389 |
| Married | 1.01 (1.01-1.01)^***^ | 1.42 (1.32-1.54)^***^ | Ref | 1.02 (0.86-1.21) | 1.53 (1.29-1.82)^***^ | 2.07 (1.71-2.50)^***^ |  |
| Other | 1.01 (1.01-1.01)^**^ | 1.34 (1.08-1.66)^**^ | Ref | 1.07 (0.62-1.85) | 2.26 (1.33-3.86)^**^ | 2.31 (1.34-3.98)^**^ |  |
| Alcohol consumption |  |  |  |  |  |  | 0.688 |
| No | 1.01 (1.01-1.01)^***^ | 1.42 (1.32-1.53)^***^ | Ref | 1.05 (0.88-1.24) | 1.61 (1.36-1.91)^***^ | 2.13 (1.77-2.57)^***^ |  |
| Yes | 1.01 (1.01-1.01)^*^ | 1.39 (1.07-1.81)^*^ | Ref | 0.81 (0.44-1.47) | 1.41 (0.77-2.57) | 1.82 (0.96-3.42) |  |
| Current smoking |  |  |  |  |  |  | 0.002 |
| No | 1.01 (1.01-1.01)^***^ | 1.59 (1.45-1.75)^***^ | Ref | 1.20 (0.98-1.47) | 1.89 (1.54-2.31)^***^ | 2.56 (2.05-3.20)^***^ |  |
| Yes | 1.01 (1.01-1.01)^***^ | 1.22 (1.09-1.36)^***^ | Ref | 0.83 (0.63-1.09) | 1.24 (0.93-1.66) | 1.53 (1.12-2.10)^**^ |  |
| Living standard |  |  |  |  |  |  | 0.186 |
| High | 1.01 (1.01-1.01) | 1.25 (0.83-1.88) | Ref | 1.71 (0.55-5.34) | 0.81 (0.26-2.50) | 1.48 (0.51-4.33) |  |
| Average | 1.01 (1.01-1.01)^***^ | 1.33 (1.20-1.47)^***^ | Ref | 1.12 (0.89-1.41) | 1.43 (1.13-1.79)^**^ | 2.12 (1.65-2.73)^***^ |  |
| Poor | 1.01 (1.01-1.01)^***^ | 1.55 (1.39-1.73)^***^ | Ref | 0.91 (0.72-1.16) | 1.92 (1.51-2.43)^***^ | 2.17 (1.67-2.84)^***^ |  |
| Educational level |  |  |  |  |  |  | 0.609 |
| Primary school or lower | 1.01 (1.01-1.01)^***^ | 1.42 (1.30-1.55)^***^ | Ref | 1.02 (0.84-1.23) | 1.77 (1.46-2.14)^***^ | 2.04 (1.64-2.53)^***^ |  |
| Middle school | 1.01 (1.01-1.01)^***^ | 1.56 (1.34-1.82)^***^ | Ref | 0.93 (0.65-1.34) | 1.07 (0.75-1.54) | 2.57 (1.77-3.73)^***^ |  |
| Senior high school or higher | 1.01 (1.01-1.01) | 1.17 (0.94-1.45) | Ref | 1.34 (0.77-2.33) | 1.82 (1.06-3.13)^*^ | 1.68 (0.90-3.14) |  |
| Hyperlipidemia |  |  |  |  |  |  | 0.301 |
| No | 1.01 (1.01-1.01)^***^ | 1.43 (1.33-1.54)^***^ | Ref | 1.02 (0.86-1.20) | 1.64 (1.39-1.93)^***^ | 2.04 (1.69-2.47)^***^ |  |
| Yes | 1.01 (1.01-1.02)^**^ | 1.65 (1.18-2.31)^**^ | Ref | 2.43 (0.84-7.00) | 1.75 (0.63-4.89) | 4.27 (1.61-11.30)^**^ |  |
| Diabetes |  |  |  |  |  |  | 0.081 |
| No | 1.01 (1.01-1.01)^***^ | 1.47 (1.36-1.59)^***^ | Ref | 1.06 (0.90-1.26) | 1.66 (1.40-1.98)^***^ | 2.14 (1.76-2.60)^***^ |  |
| Yes | 1.01 (1.01-1.01) | 1.12 (0.92-1.35) | Ref | 0.67 (0.40-1.11) | 0.94 (0.59-1.49) | 1.32 (0.80-2.19) |  |
| CKD |  |  |  |  |  |  | 0.557 |
| No | 1.01 (1.01-1.01)^***^ | 1.48 (1.34-1.63)^***^ | Ref | 1.07 (0.85-1.34) | 1.46 (1.17-1.83)^***^ | 2.27 (1.79-2.88)^***^ |  |
| Yes | 1.01 (1.01-1.01)^*^ | 1.29 (1.01-1.66)^*^ | Ref | 1.08 (0.57-2.05) | 2.03 (1.04-3.97)^*^ | 1.49 (0.71-3.09) |  |
| CVD |  |  |  |  |  |  | 0.481 |
| No | 1.01 (1.01-1.01)^***^ | 1.41 (1.30-1.52)^***^ | Ref | 1.02 (0.87-1.21) | 1.53 (1.29-1.81)^***^ | 2.08 (1.72-2.52)^***^ |  |
| Yes | 1.01 (1.01-1.01)^**^ | 1.46 (1.16-1.83)^**^ | Ref | 1.14 (0.64-2.05) | 2.41 (1.39-4.20)^**^ | 2.26 (1.29-3.96)^**^ |  |

^*^*P* values <0.05; ^**^*P* values <0.01; ^***^*P* values <0.001.


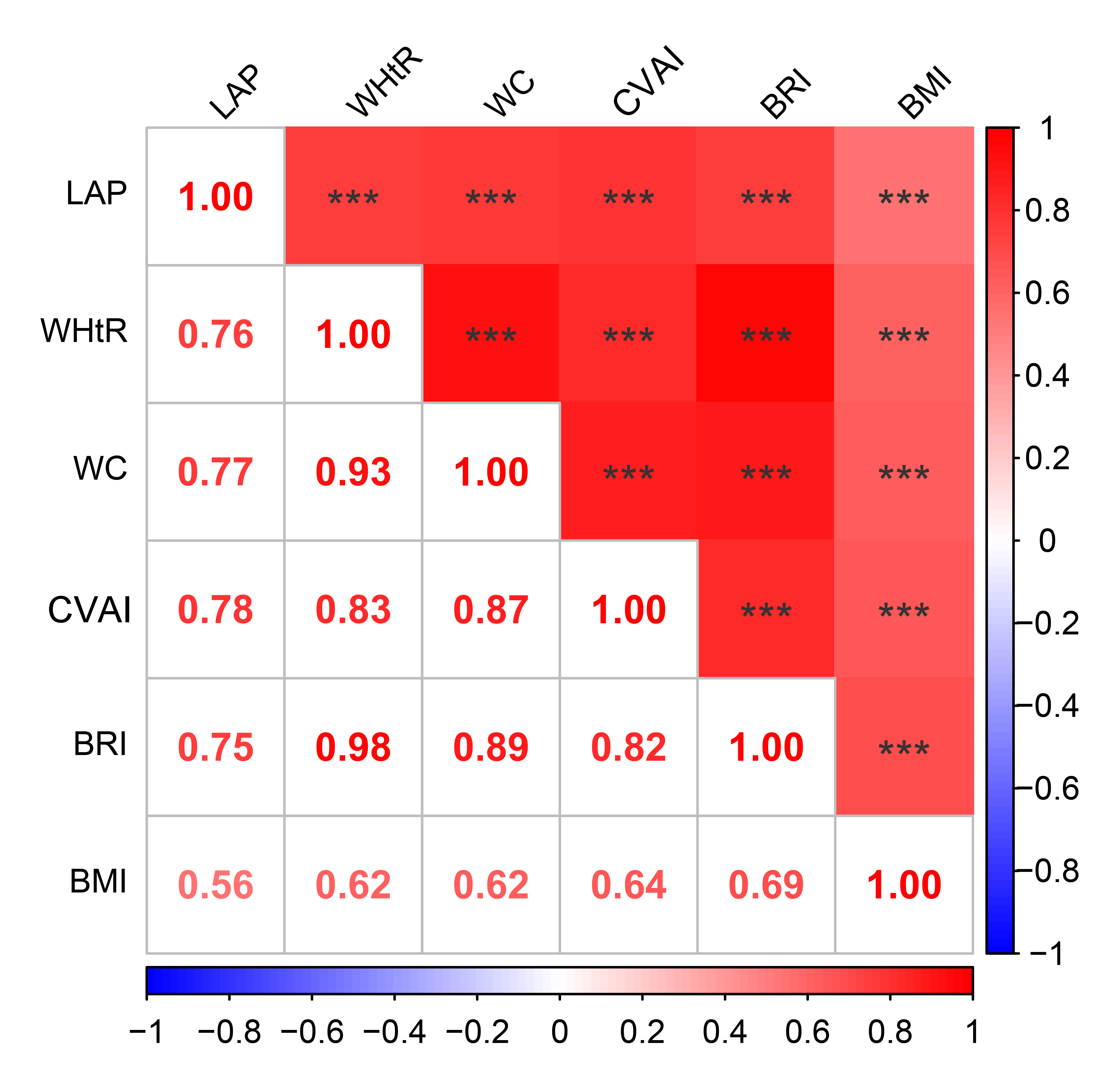


**Supplementary Fig. S1 The correlation between anthropometric indices**


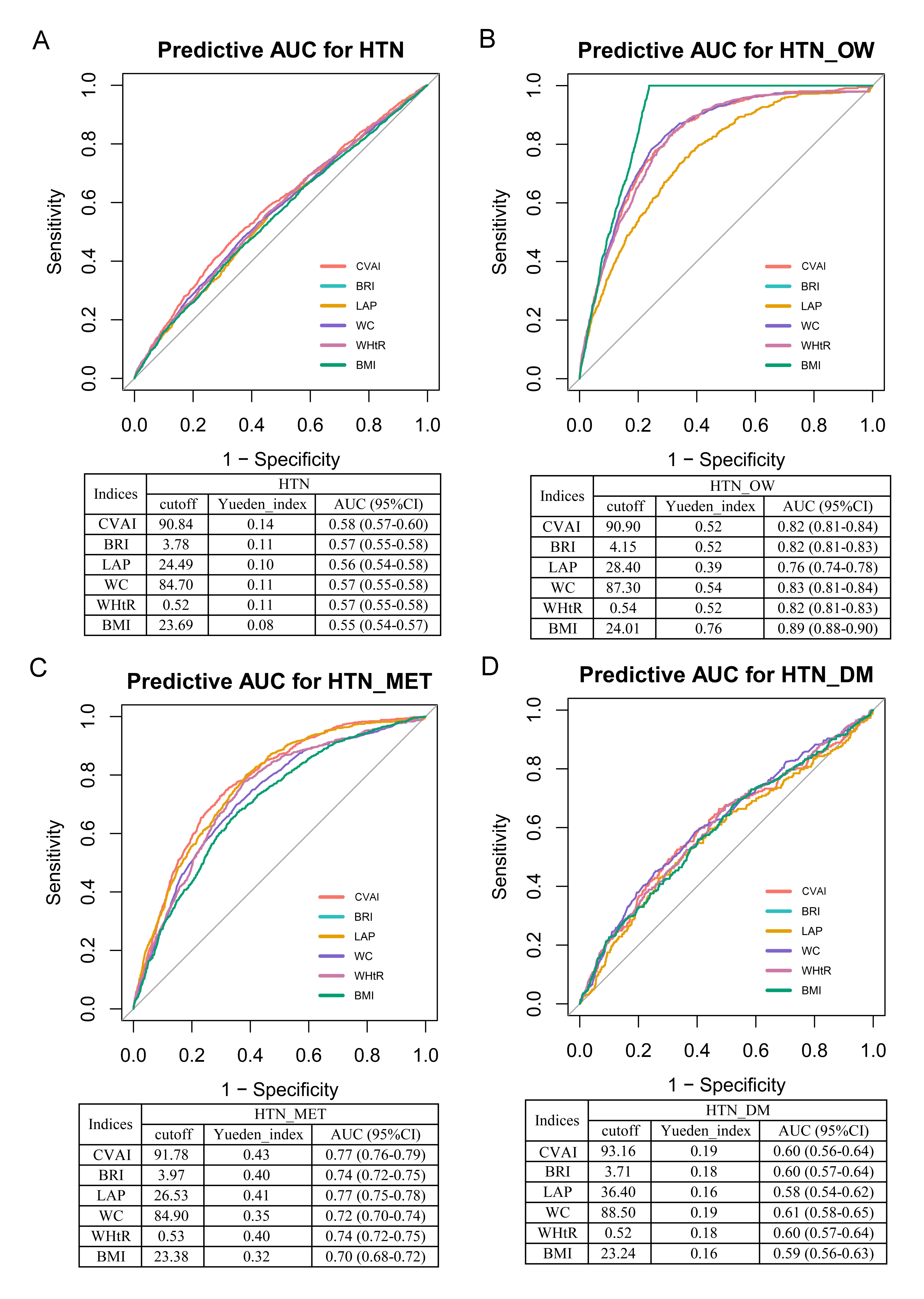


**Supplementary Fig. S2 The ROC curves and area under the curves (AUCs) (95% CIs) of baseline CVAI for predicting the risk of hypertension and comorbidity.**

A. The predictive ROC curves of baseline CVAI for predicting the risk of HTN.

B. The predictive ROC curves of baseline CVAI for predicting the risk of HTN-OW.

C. The predictive ROC curves of baseline CVAI for predicting the risk of HTN-MET.

D. The predictive ROC curves of baseline CVAI for predicting the risk of HTN-DM.

Abbreviation: CVAI, Chinese visceral adiposity index; HTN, hypertension; HTN-OW, hypertension and overweight comorbidity; HTN-MET, hypertension and metabolic disorder comorbidity; HTN-DM, hypertension and diabetes comorbidity; ROC, receiver operator characteristic curve.


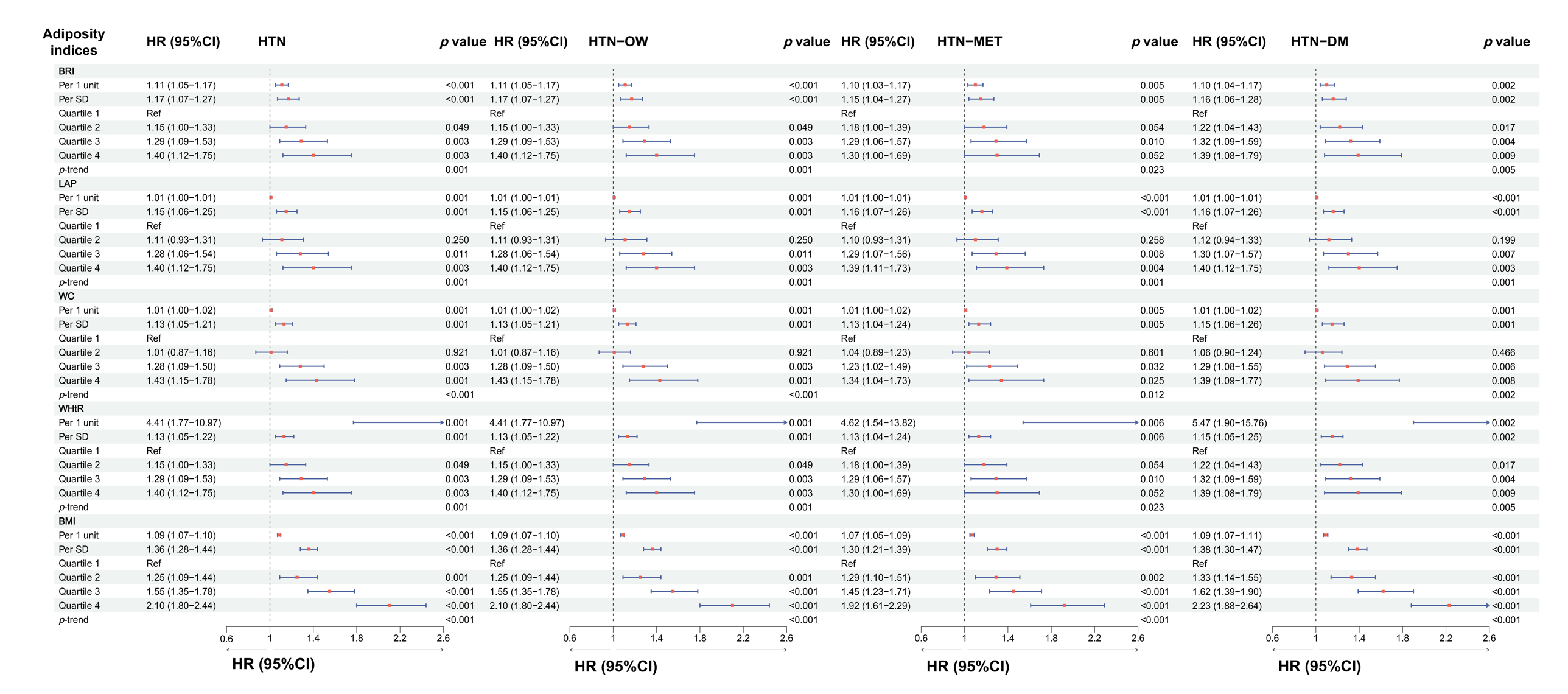


**Supplementary Fig. S3 Forest plot of the association between other adiposity indices and risk of hypertension and comorbidity.**

For BRI, LAP, WC, WHtR study models, adjusted for sex, age, BMI, education, marital status, residence, living standard, smoking status, drinking status, uric acid, serum creatinine, FPG, history of dyslipidemia, diabetes, CVD, liver disease, kidney disease, chronic lung diseases at baseline. For the BMI model adjusted for all covariates except BMI.

**Reference**

1. Qu L, Fang S, Lan Z, Xu S, Jiang J, Pan Y, et al., Association between atherogenic index of plasma and new-onset stroke in individuals with different glucose metabolism status: insights from CHARLS. Cardiovasc Diabetol 23 (2024) 215.dio: 10.1186/s12933-024-02314-y

2. Han M, Qin P, Li Q, Qie R, Liu L, Zhao Y, et al., Chinese visceral adiposity index: A reliable indicator of visceral fat function associated with risk of type 2 diabetes. Diabetes Metab Res Rev 37 (2021) e3370.dio: 10.1002/dmrr.3370

3. Amato MC, Giordano C, Galia M, Criscimanna A, Vitabile S, Midiri M, et al., Visceral Adiposity Index: a reliable indicator of visceral fat function associated with cardiometabolic risk. Diabetes Care 33 (2010) 920-2.dio: 10.2337/dc09-1825

4. Thomas DM, Bredlau C, Bosy-Westphal A, Mueller M, Shen W, Gallagher D, et al., Relationships between body roundness with body fat and visceral adipose tissue emerging from a new geometrical model. Obesity (Silver Spring) 21 (2013) 2264-71.dio: 10.1002/oby.20408

5. Kahn HS, The "lipid accumulation product" performs better than the body mass index for recognizing cardiovascular risk: a population-based comparison. BMC Cardiovasc Disord 5 (2005) 26.dio: 10.1186/1471-2261-5-26

6. Khamseh ME, Malek M, Abbasi R, Taheri H, Lahouti M, and Alaei-Shahmiri F, Triglyceride Glucose Index and Related Parameters (Triglyceride Glucose-Body Mass Index and Triglyceride Glucose-Waist Circumference) Identify Nonalcoholic Fatty Liver and Liver Fibrosis in Individuals with Overweight/Obesity. Metab Syndr Relat Disord 19 (2021) 167-173.dio: 10.1089/met.2020.0109

7. Malek M, Khamseh ME, Chehrehgosha H, Nobarani S, and Alaei-Shahmiri F, Triglyceride glucose-waist to height ratio: a novel and effective marker for identifying hepatic steatosis in individuals with type 2 diabetes mellitus. Endocrine 74 (2021) 538-545.dio: 10.1007/s12020-021-02815-w

8. Wang X, Liu J, Cheng Z, Zhong Y, Chen X, and Song W, Triglyceride glucose-body mass index and the risk of diabetes: a general population-based cohort study. Lipids Health Dis 20 (2021) 99.dio: 10.1186/s12944-021-01532-7

9. Zhang S, Du T, Zhang J, Lu H, Lin X, Xie J, et al., The triglyceride and glucose index (TyG) is an effective biomarker to identify nonalcoholic fatty liver disease. Lipids Health Dis 16 (2017) 15.dio: 10.1186/s12944-017-0409-6
